# Evolution of Antibiotic Resistance in *Escherichia coli* and *Klebsiella pneumoniae* Clinical Isolates in a Multi-Profile Hospital over 5 Years (2017–2021)

**DOI:** 10.3390/jcm12062414

**Published:** 2023-03-21

**Authors:** Beata Mączyńska, Magdalena Frej-Mądrzak, Jolanta Sarowska, Krystyna Woronowicz, Irena Choroszy-Król, Agnieszka Jama-Kmiecik

**Affiliations:** 1 Department of Pharmaceutical Microbiology and Parasitology, Faculty of Pharmacy, Medical University, 50-367 Wroclaw, Poland; 2 Department of Hygiene and Epidemiology, Lower Silesian T. Marciniak Specialist Hospital-Center for Emergency Medicine, 54-049 Wrocław, Poland; 3 Department of Basic Sciences, Faculty of Health Sciences, Medical University, 50-367 Wroclaw, Poland; 4 Medical Laboratory Synevo, Fieldorfa 2, 50-049 Wroclaw, Poland

**Keywords:** multi-drug resistant strains, *Enterobacteriaceae*, ESBL

## Abstract

In recent years, we have witnessed a growing drug resistance among bacteria, which is associated with the use and availability of an increasing number of broad-spectrum antimicrobial agents, as well as with their irrational and excessive use. The present study aims to analyze changes in the drug resistance of Gram-negative *Enterobacterales*: *Escherichia coli* and *Klebsiella pneumoniae*, isolated from infections in a multi-profile hospital over five years (from 2017 to 2021). Among the practical outcomes of the evaluation of these data will be the possibility of determining changes in susceptibility to the antibiotics used in the hospital. In turn, this will help propose new therapeutic options, especially for empirical therapy that is necessary in severe infections. The analysis of the use of individual groups of antibiotics allowed for identification of the causes of the increasing resistance of Gram-negative bacilli. The highest number of infections whose etiological agent was *K. pneumoniae* ESBL(+) and *E. coli* ESBL(+) was observed in 2018. In the analyzed five-year period, the number of multi-resistant (MDR) *K. pneumoniae* strains increased successively, which seems to be related to the growing use, especially in the pandemic period, of broad-spectrum antibiotics, mainly penicillins with inhibitors, third-generation cephalosporins, and carbapenems.

## 1. Introduction

Optimization of antibiotic use involves choosing antimicrobial agents that have the weakest possible effect on the microbiota and a low risk of inducing bacterial resistance [1]. Targeted antibiotic therapy is the best therapeutic option, but it requires identification of the pathogen causing the infection and determination of its antibiotic susceptibility, for which there is not always time during in-hospital treatment. In the case of a life-threatening condition of the patient, empirical therapy is used, which involves the selection of a drug, taking into account, among other things, the site of infection or the presence of inflammatory markers, as well as the current epidemiological situation in the geographical area or even in a particular hospital [2]. Unfortunately, recent years have witnessed growing drug resistance among bacteria, which is associated with the use and availability of an increasing number of broad-spectrum antimicrobial agents, as well as with their irrational and excessive use. The above is a very dangerous phenomenon, as it results in a loss of therapeutic options and, consequently, in a decreased control over infections [3].

Extended-spectrum β-lactamases (ESBLs) are the real challenge faced by modern medicine. They have the ability to inactivate both penicillins and first-, second- and third-generation cephalosporins (in addition to cephamycins), as well as monobactams, thus significantly complicating antibiotic therapy [4]. Bacterial strains showing production of extended-spectrum β-lactamases are included in the list of alert pathogens and *E. coli* ESBL(+) has been recognized as one of the alert pathogens of greatest importance [5,6]. *E. coli* also shows resistance to quinolones. To make things worse, it is associated with plasmid transmission of e.g., *gyr* genes, which is very often simultaneously observed with ESBL [7]. In the case of the increasingly prevalent chromosomally encoded resistance, the most common mechanisms are a change in target enzyme (gyrase and/or topoisomerase IV) conformation resulting in an up to 10-fold decrease in their activity, as well as increased efflux pump activity, causing the drug to be actively pumped out of the bacterial cell. Plasmid-encoded resistance mechanisms also include Qnr, QepA, and OqxAB proteins. Qnr proteins responsible for altering the target site of antibiotic action compete with DNA in the formation of the gyrase-DNA-quinolone complex, resulting in the reduced susceptibility of *E. coli* to this group of antibiotics. The QepA and OqxAB proteins, on the other hand, are a type of membrane pumps that allow the antibiotic to be actively shed from inside the cell [8]. In the case of aminoglycosides, alteration of the target site of action, which is the 16S rRNA of the 30S subunit of the ribosome, by plasmid-translocated methyltransferases, prevents the drug from binding to the ribosome, and thus leads to the lack of translation inhibition and to the development of resistance to this group of antibiotics [9]. In turn, the evolution of *K. pneumoniae* resistance exemplifies one of the most dynamic evolutions among bacteria in recent years [6,10]. These bacilli have resistance genes that can be present in chromosomes, but also in plasmids’ or transposons’ conditioning natural resistance to glycopeptides or phenoxypenicillins. Any disruption of their structure can result in an expansion of the spectrum of inactivated antibiotics, as drug-resistant microorganisms in the hospital environment undergo positive selection [11]. As in the case of *E. coli*, resistance to other groups of antibiotics, such as quinolones and aminoglycosides, also occurs in *K. pneumoniae* along with ESBLs. Until recently, in Poland, TEM and CTX-M families have dominated, while enzymes of the OXA family, which very often are not even included in ESBLs due to their failure to hydrolyze third-generation cephalosporins, have been much less common [12]. These β-lactamases condition resistance (quite weak) to cefotaxime, ceftriaxone, and aztreonam only, while enzymes of the PER family have a special ability to hydrolyze many cephalosporins [13,14].

The most dangerous situation is related to the development of resistance to all antibiotics belonging to the β-lactam group through the coexistence of AmpC/ESBL and disruption of porin protein expression [15]. In the course of the evolution of β-lactamases, enzymes specializing in the hydrolysis of carbapenems were also developed [3]. *K. pneumoniae* carbapenemases (KPC) fall into three classes: A, B, and D. Class B carbapenemases, or metallo-β-lactamases (MBLs), are characterized by their ability to hydrolyze penicillins, cephalosporins, and carbapenems, and they lack the ability to hydrolyze monobactams. Group A carbapenemases with high clinical relevance and the widest substrate spectrum include enzymes from the KPC family [4,6]. They have the ability to hydrolyze all β-lactams, and it so happens that strains remain resistant to all available antibiotics (PDR Pan-Drug Resistance) [16]. *K. pneumoniae* KPC+ infections are characterized by a very severe course with a high risk of patient death. The rapid spread of these pathogens in the environment poses an additional problem [17]. D-type carbapenemases, or CHDLs, one of which is OXA-48, are characterized by resistance to temocillin and a lack of susceptibility to EDTA and clavulanic acid. Depending on the locus, OXA-48(+) strains show resistance to all β-lactam antibiotics or a reduced susceptibility to carbapenems with preserved susceptibility to third-generation cephalosporins and monobactams [18]. In addition to resistance to β-lactam antibiotics, *K. pneumoniae* bacilli have also developed an enzymatic and receptor-mediated mechanism of resistance to aminoglycosides [19]. In addition, through a transport mechanism, *Klebsiella* have developed a resistance mechanism which actively removes the antibiotic from the cells via membrane pumps. In resistant strains, the number of efflux pumps in the cell membrane increases, leading to insusceptibility not only to quinolones, but also to aminoglycosides and β-lactams [20]. The present study aims to analyze changes in drug resistance in Gram-negative *Enterobacterales*: *E. coli* and *K. pneumoniae*, isolated from infections in a multi-profile hospital over five years (from 2017 to 2021). Among the practical outcomes of the evaluation of these data will be the possibility of determining changes in susceptibility to the antibiotics used in the hospital, which will help in developing new therapeutic options, especially for empirical therapy that is necessary in severe infections. In turn, analysis of the consumption of individual groups of antibiotics may allow us to identify the causes of the increasing resistance of Gram-negative bacilli. We hope to detect new trends in the resistance of these microorganisms and improve the effectiveness of patient therapy.

## 2. Materials and Methods

### 2.1. Bacterial Strains

All analyzed data came from the information and materials obtained from a multi-profile hospital in Wroclaw and cover the period from 1 January 2017 to 31 December 2021. The materials were obtained from hospital patients as part of routine microbiological tests. Annually, an average of 11,920 microbiological tests are performed at the selected institution, of which an average of 3561 (about 30%) turn out to be positive. In order to observe changes in the drug susceptibility of bacteria isolated from patients of the multi-profile hospital in Wroclaw, 2 species were selected that were the most relevant from the point of view of drug resistance. The development of drug resistance of bacteria from the following species was analyzed: *E. coli* (strains were divided into ESBL(+) and ESBL(−), *K. pneumoniae* (strains were divided into ESBL(+), and ESBL(−)). These strains were mainly isolated from urinary tract infections and pneumonia. Their numbers for 2017–2021 are shown in Table 1. Each year, the frequency of *E. coli* infections was significantly higher than the frequency of *K. pneumoniae* (Table 1)

Depending on the strain, resistance to specific selected antibiotics was analyzed.

### 2.2. Microbiological Tests

#### 2.2.1. Automatic Systems

The study was carried out according to the following scheme: from the identification of strains through drug resistance analysis to the detection of carbapenemases by enzymatic and disk diffusion methods [21]. To identify the microorganisms, we isolated the strains from patient materials on appropriate microbiological media. Identification was made by evaluating colony morphology on plates and then using the VITEK^®^2 system (bioMerieux, Craponne, France). This system makes it possible not only to confirm identification of species, but also to perform antibiograms. It uses a bacterial suspension with a density of 0.5 McFarland, as well as appropriate identification and antibiogram cards. The results were issued on the basis of biochemical and enzymatic tests performed to distinguish between the different strains. The corresponding VITEK^®^2 identification cards (AST-N331, AST-N332, GNID for different microorganisms) are placed in the VITEK^®^2 apparatus (bioMerieux, France), and a computer program identifies and evaluates the drug susceptibility of the strains. Two types of antibiogram cards for Gram-negative bacteria were used to assess drug susceptibility using the VITEK2 system. These were VITEK2 AST-331 cards (amikacin, ampicillin/sulbactam, aztreonam, cefepime, ceftazidime, ciprofloxacin, colistin, gentamicin, imipenem, levofloxacin, meropenem, piperacillin, piperacillin/tazobactam, ticarcillin/clavulanic acid, tobramycin, trimethoprim/sulfur) and VITEK2 AST-332 (amikacin, amoxicillin/clavulanic acid, cefepime, cefotaxime, ceftazidime, cefuroxime, ciprofloxacin, colistin, ESBL confirmation, gentamicin, imipenem, meropenem, piperacillin/tazobactam, tigecycline, tobramycin, trimethoprim/sulfur) (Table 2). The use of this system made it possible to determine MIC values.

For some antibiotics absent from the VITEK panels, bacterial susceptibility was also tested using the drug concentration gradient strip diffusion method (E-tests). The results obtained were interpreted according to EUCAST recommendations [22,23].

#### 2.2.2. The Disk Diffusion Method

The disk diffusion method was used to identify extended-spectrum β-lactamases (ESBLs). In this method, discs with ceftazidime and cefotaxime are used, arranged at a distance of 2 cm (between the centers) from the disc with amoxicillin-clavulanic acid. A positive test result consists of a marked enlargement of the zone of inhibition around the disc with ceftazidime or cefotaxime (cefpodoxime, aztreonam) on the side of the disc containing clavulanic acid. This enlargement can take very different shapes [24,25].

At the same time, disc methods were also used to identify the type of carbapenemases. For the phenotypic test detecting KPC-class carbapenemases, a 10-μg meropenem disc and a 10-μg meropenem disc soaked in boronic acid were used, maintaining a minimum distance of 3 cm between them, and the plate was incubated at 35 °C for 18 h. A positive result for *Enterobacteriaceae* is indicated by a difference of ≥4 mm in zones of inhibition between MEM10+KB and MEM10. In *Pseudomonas* spp. and *Acinetobacter* spp. on the other hand, a difference in zones of inhibition of ≥7 mm is considered a positive result. For the detection of MBL-class carbapenemases, sterile discs soaked in EDTA solution and discs with 30 μg ceftazidime and 10 μg imipenem were used, maintaining a gap of 2 cm between them [26]. An enlargement of the zone of inhibition around the disc with CAZ30 and/or IMP10 toward the disc with EDTA is considered to be a positive result. Detection of OXA-48 carbapenemases is possible using a disc with 30 μg temocillin [24,25]. A reduction of ≤10 mm in the zone of inhibition around the TEM30 disc is then observed.

#### 2.2.3. Enzyme and Immunochromatographic Tests

The CarbaNP test (Argenta) allows for the detection of carbapenemases in *Enterobacteriaceae*, but without determining their type. It is rapid (2 h) and is based on the color change of the indicator from red to yellow. This is only possible if the test strain produces enzymes that hydrolyze the carbapenem (imipenem) contained in the test. This is because the above hydrolysis leads to a decrease in the pH of the environment and to a resulting change in the color of the indicator. The difference in color in the test and control tubes indicates a positive test (carbapenemase production). The test was performed in accordance with the recommendations of KORLD [27]. Resist O.O.K.N.V. immunochromatographic assays have also been used to detect carbapenem-hydrolyzing enzymes in *Enterobacteriaceae*. They allow for the detection of carbapenemases such as KPC, OXA-163, OXA-48, NDM, and VIM [27].

### 2.3. Statistical Analysis

The variables were expressed as a frequency: as an absolute value and as a percentage. Chi-square tests were used to compare categorical variables. The Mann–Whitney test was used to compare two different strains. The Kruskal–Wallis H test assessed the differences between strain resistance over 5 years. To explore differences in pairs, Bonferroni–Dunn post hoc tests were used. Two-sided *p*-value < 0.05 indicated statistical significance. Statistical analyses were performed using PQStat version 1.8.0.

## 3. Results

### 3.1. Evolution of Resistance of Selected Bacteria to Antibiotics Used in Therapy

#### 3.1.1. Antibiotic Resistance of *E. coli* Strains from 2017 to 2021

The annual number of infections in the analyzed hospital is at a relatively constant level and ranges from 1012 to 1321, with the highest number in 2018 and the lowest in 2020. Each year, the highest number of infections was caused by *E. coli*.

In the case of *E. coli*, the resistance of strains producing extended-spectrum β-lactamases (ESBL(+)) and those lacking this ability (ESBL(−)) were considered separately.

**Figure 1:**
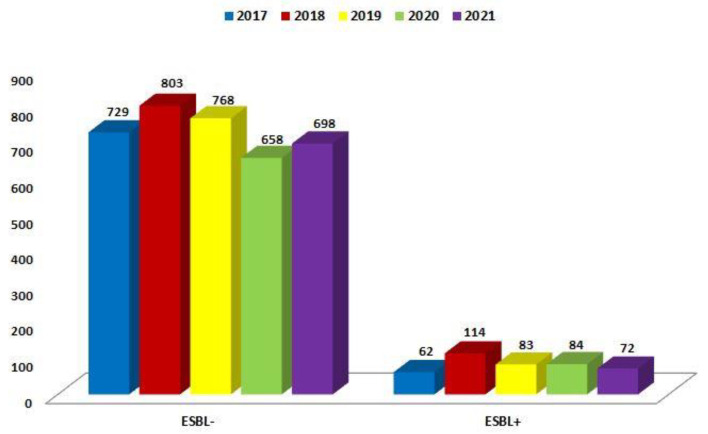
Comparison of the number of *E. coli* ESBL(+) and ESBL(−) strains in 2017–2021.

Over the past five years, the number of ESBL(+) and ESBL(−) strains has been relatively constant. For ESBL(−) strains, it ranged from 658 (88%; 2020) to 803 (87%; 2018), while for ESBL(+) strains, it ranged from 62 (8%; 2017) to 114 (12%; 2018). The highest number of infections caused by *E. coli* was recorded in 2018. Each year, the ESBL(−) variant, which does not produce extended-spectrum β-lactamases, dominated amongst *E. coli* (Figure 1).

**Figure 2:**
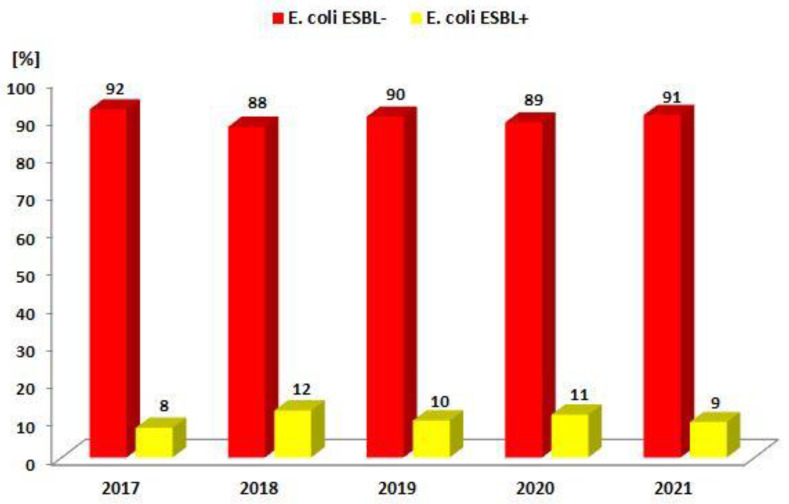
Comparison of the percentage of *E. coli* ESBL(+) and ESBL(−) strains from 2017 to 2021.

Each year, the percentage of ESBL(−) strains was almost 10 times higher than ESBL(+) strains and ranged from 88% to 92% over the past five years (Figure 2, Table 3). It was significantly higher than the frequency of *E. coli* ESBL(+) infections (Table 3). The percentage of ESBL(+) strains, on the other hand, ranged from 8% to 12% and did not increase significantly for *E. coli*. However, it significantly differs in particular years (*p* < 0.05)—there is a higher observed frequency of ESBL(−) strains in 2017 and ESBL(+) strains in 2018 than expected.

Due to the natural resistance of *E. coli* to penicillin G, only the resistance of strains to semisynthetic penicillins (ampicillin) and penicillins with inhibitors was analyzed.

Among *E. coli* ESBL(−) strains, relatively, there was the highest resistance to ampicillin, i.e., from n = 377 (54%; 2021) to n = 538 (67%; 2018). In addition, lower resistance of these strains to amoxicillin with clavulanic acid was observed from n = 197, n = 230 (which is 30%; 2018, 2019 respectively) to n = 343 (47%; 2017) and slightly higher resistance to ampicillin with sulbactam from n = 328 (47%; 2021) to n = 375–458 depending on the year (57%; 2018–2020). *E. coli* ESBL(−) strains showed the greatest susceptibility (over 85%) to piperacillin with tazobactam. For *E. coli* ESBL(−) strains, when comparing resistance to penicillins (ampicillin) and penicillins with inhibitors in each year separately, resistance significantly differs in each year (*p* < 0.001). In-pairs comparisons (post-hoc) show that in every year, there was a significant difference between resistance to each ampicillin vs. piperacillin with tazobactam (*p* < 0.001). In 2017, there were no significant differences between other pairs. In 2018, a significant difference between ampicillin and amoxicillin with clavulanic acid (*p* < 0.01) was noticed. In 2019 and 2020, there was a significantly better response from ampicillin compared with amoxicillin with clavulanic acid. In 2021, similarly, ampicillin differs significantly to amoxicillin with clavulanic acid (*p* < 0.001).

**Figure 3:**
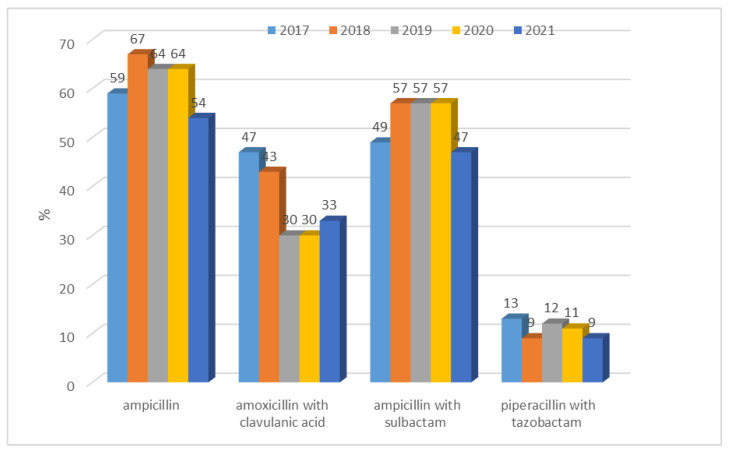
Percentage of *E. coli* ESBL(−) strains resistant to penicillins (ampicillin) and penicillins with inhibitors.

**Figure 4:**
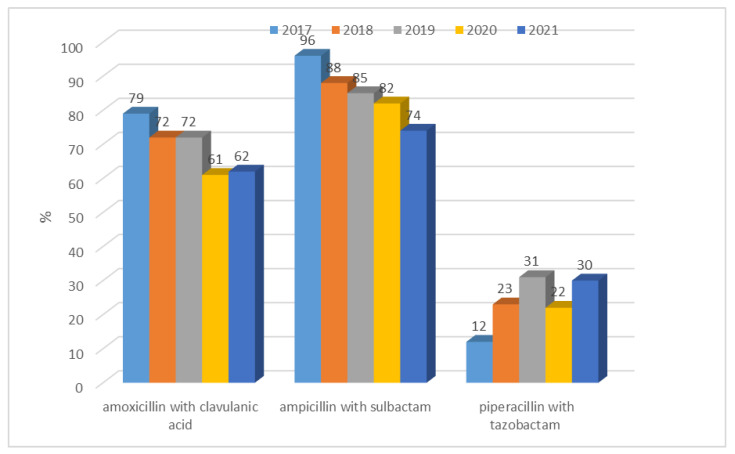
Percentage of *E. coli* ESBL(+) strains resistant to penicillins with inhibitors.

Among *E. coli* ESBL(−) strains, resistance to penicillins was lower in each case than for ESBL(+) strains (Figure 3 and Figure 4). The difference was significant for each penicillin with inhibitors: amoxicillin with clavulanic acid (*p* < 0.001), ampicillin with sulbactam (*p* < 0.001), and piperacillin with tazobactam (*p* < 0.001).

*E. coli* ESBL(+) were completely resistant to amoxicillin, and resistance to penicillins with inhibitors was significantly higher than in bacilli, which do not produce extended-spectrum β-lactamases. Comparing resistance to *E. coli* ESBL(+) and *E. coli* ESBL(−) for amoxicillin with clavulanic acid, differences were significant for each year: 2017 *p* < 0.001, 2018 *p* < 0.001, 2019 *p* < 0.001, 2020 *p* < 0.001, 2021 *p* < 0.001.

Comparing resistance to *E. coli* ESBL(+) and *E. coli* ESBL(−) for ampicillin with sulbactam 2017, differences were significant for each year: 2017 *p* < 0.001, 2018 *p* < 0.001, 2019 *p* < 0.001, 2020 *p* < 0.001, 2021 *p* < 0.001.

Comparing resistance to *E. coli* ESBL(+) and *E. coli* ESBL(−) for piperacillin with tazobactam, there were significant differences in 2018: *p* < 0.05, 2019 *p* < 0.001, 2020 *p* < 0.05, 2021 *p* < 0.001. There was no significant difference in 2017.

This ranged from n = 51 (61%; 2020) to n = 49 (79%; 2017) for amoxicillin with clavulanic acid, from n = 53 (74%) to n = 60 (96%) for ampicillin with sulbactam, and from n = 7 (12%; 2017) to n = 26 (31%; 2019) for piperacillin with tazobactam. *E. coli* ESBL(+) strains were most susceptible to piperacillin with tazobactam, and this trend continued for all the analyzed years.

Resistance of *E. coli* to other groups of antibiotics such as cephalosporins, carbapenems, aminoglycosides, and quinolones was also considered. There was no resistance of *E. coli* ESBL(+) and ESBL(−) strains to carbapenems over a five-year period at the selected hospital, and all *E. coli* showed 100% susceptibility to meropenem and imipenem.

**Figure 5:**
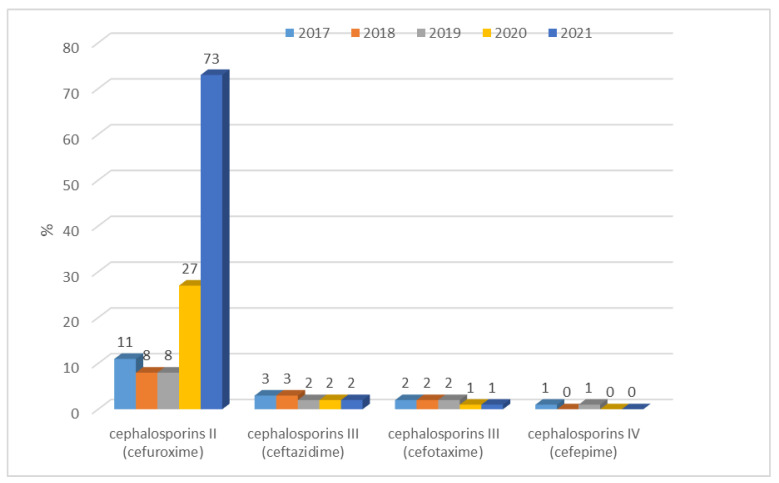
Percentage of cephalosporin-resistant *E. coli* ESBL(−) strains.

**Figure 6:**
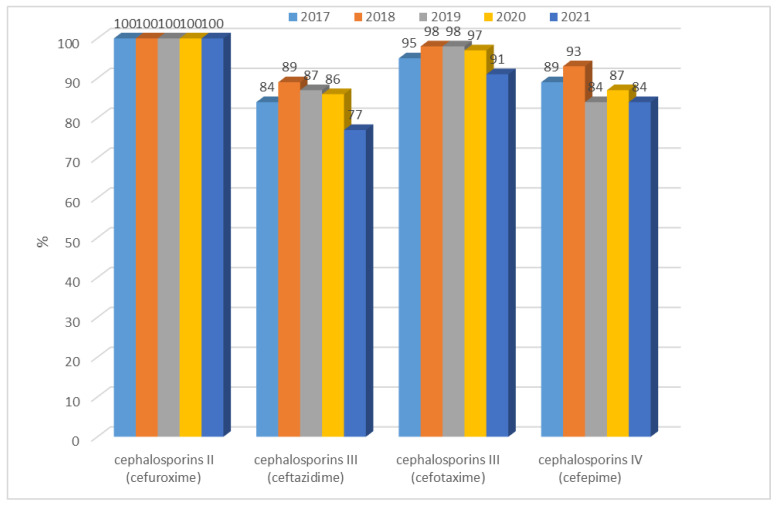
Percentage of *E. coli* ESBL(+) strains resistant to cephalosporins.

*E. coli* strains not producing extended-spectrum β-lactamases only showed resistance to cefuroxime (second-generation cephalosporins). Then, rapid significant (*p* < 0.001) changes were reported: in 2020, it more than doubled to 27% (n = 178) and during the last year, it reached 73% (n = 510), an almost 3-fold increase. Until 2019, it did not exceed 11% (n = 80), while in 2020, it more than doubled to 27% (n = 178), and during the last year, it reached 73% (n = 510), an almost 3-fold increase. Resistance over five years of ESBL(−) strains to cephalosporins III and IV proved to be very low, ranging from 1% to 3% (Figure 5). In contrast, a much higher resistance to cephalosporins was observed in *E. coli* ESBL(+) strains. These bacteria showed a complete lack of susceptibility to second-generation cephalosporins and a very high resistance (above 80%) to third- and fourth-generation cephalosporins. The highest number of susceptible strains was recorded for ceftazidime. Over the past year, there has been a slight increase in the susceptibility of these bacilli to cefotaxime, ceftazidime, and cefepime (Figure 6). Increases in the susceptibility of these bacilli to cefotaxime, ceftazidime, and cefepime were observed, however were no significant differences when comparing year-to-year changes.

**Figure 7:**
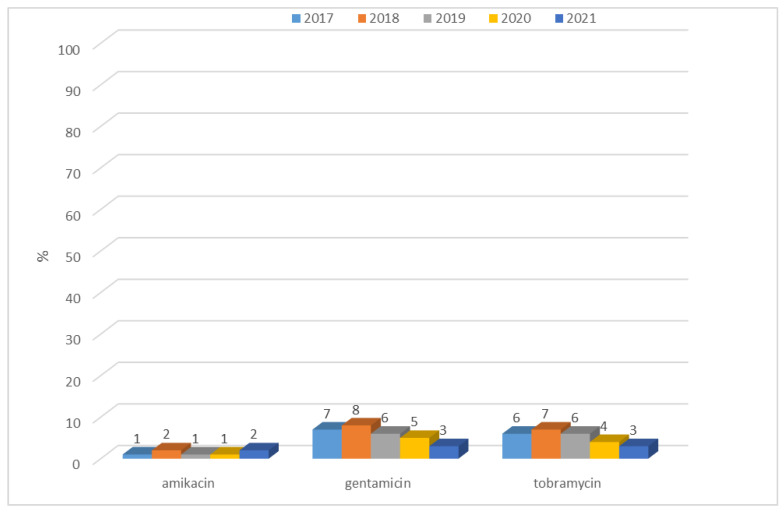
Percentage of aminoglycoside-resistant *E. coli* ESBL(−) strains.

**Figure 8:**
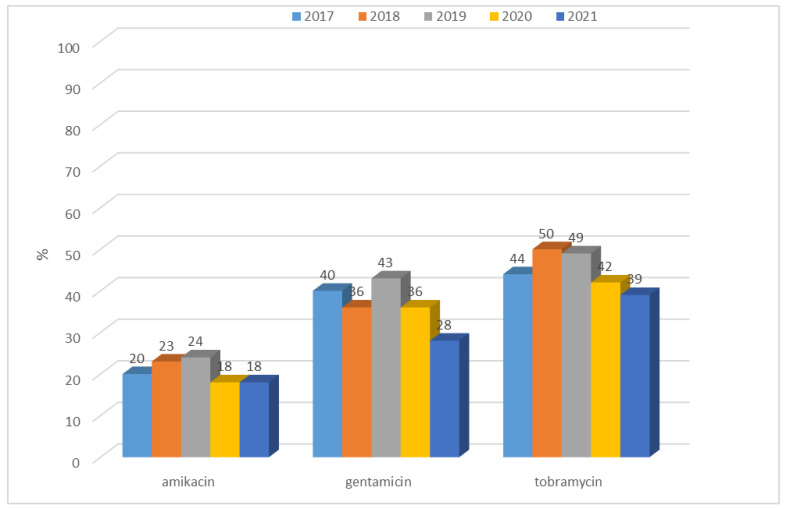
Percentage of *E. coli* ESBL(+) strains resistant to aminoglycosides.

Among ESBL(−) bacilli, resistance to aminoglycosides was low, from 1% to 8%, however, changes in resistance to amikacin and gentamicin are significant (respectively *p* < 0.05 and *p* < 0.01). The highest percentage of resistant strains was recorded in 2018. In contrast, for *E. coli* producing extended-spectrum β-lactamases, resistance to aminoglycosides was at a much higher level, ranging from 18% (n = 13; 2020 and 2021) to 50% (n = 57; 2018) (Figure 7). Each year, for each type of aminoglycosides, differences between responses to ESBL(−) and ESBL(+) was significant (*p* < 0.001). More than half of the *E. coli* ESBL(+) strains remained susceptible to aminoglycosides. The highest percentage of resistant strains was reported in 2019 (amikacin, gentamicin) and there was even a slight increase in susceptibility to this group of antibiotics over the past two years (Figure 8). However, observed changes in year-to-year responses to amikacin, gentamicin, and tobramycin are not significant. The highest susceptibility was recorded to amikacin, both among ESBL(+) and ESBL(−) strains.

**Figure 9:**
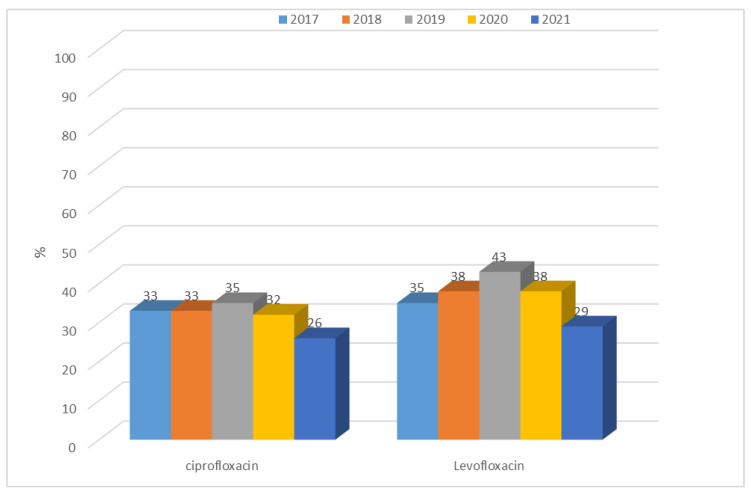
Percentage of quinolone-resistant *E. coli* ESBL(−) strains.

**Figure 10:**
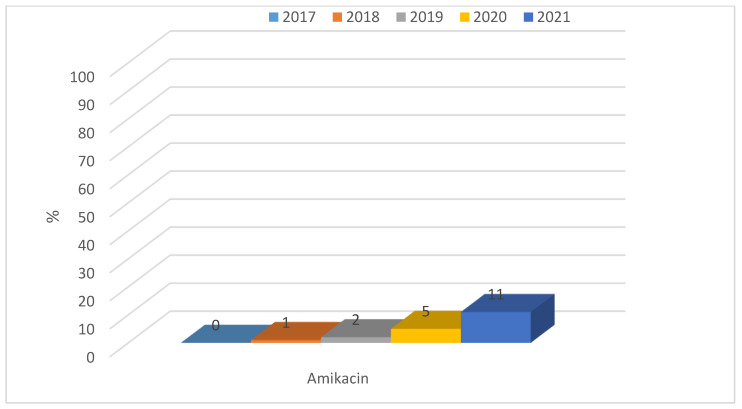
Percentage of quinolone-resistant *E. coli* ESBL(+) strains.

Over the past five years, the quinolone resistance of *E. coli* ESBL(−) strains has been ranging from 26% (n = 181; 2021) to 43% (n = 330; 2019) (Figure 9). The differences in the resistance of *E. coli* ESBL(−) strains to quinolone year-to-year are significant (ciprofloxacin *p* < 0.01, levofloxacin *p* < 0.001). For ciprofloxacin, the resistance was significantly lower in 2021 compared with 2017–2019 (*p* < 0.05, *p* < 0.05, *p* < 0.01, respectively). For levofloxacin, the resistance was significantly higher in 2019 compared with 2017 (*p* < 0.05) and was significantly lower in 2021 compared with 2018–2020 (*p* < 0.01, *p* < 0.001, *p* < 0.01, respectively) Resistance of strains producing extended-spectrum β-lactamases to quinolones was at a much higher level compared with ESBL(−) strains, ranging from 78% (n = 56; 2021) to 91% (n = 104; 2018). The highest resistance to quinolones was recorded in 2018 and the lowest in 2021. Still, the resistance was very high, despite the decreasing trend observed in the last three years (Figure 10). The differences in the resistance of *E. coli* ESBL(+) strains to quinolone year-to-year are not significant.

#### 3.1.2. Antibiotic Resistance of *K. pneumoniae* Strains in 2017–2021

When analyzing changes in the antibiotic resistance of *K. pneumoniae* bacilli, a division was applied (as for *E. coli*) between strains having the ability to produce extended-spectrum β-lactamases (ESBL(+)) and those not producing these enzymes—ESBL(−). Resistance of these bacilli to such antibiotic groups as penicillins with inhibitors, cephalosporins, carbapenems, aminoglycosides, and quinolones was considered.

**Figure 11:**
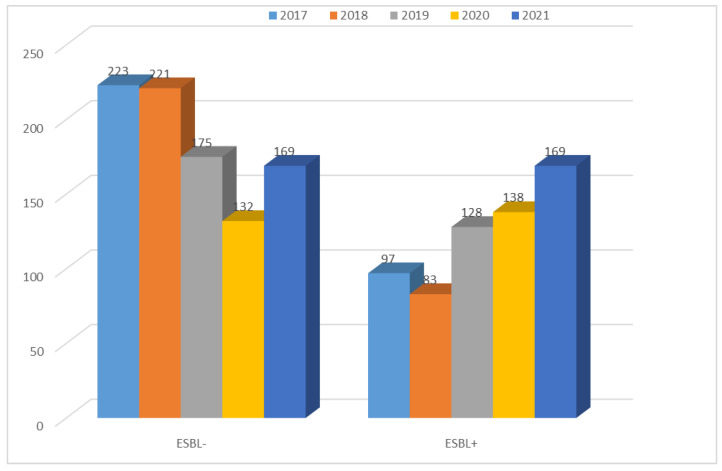
Comparison of the number of *K. pneumoniae* ESBL(+) and ESBL(−) strains from 2017 to 2021.

The number of β-lactamase-producing *K. pneumoniae* strains ranged from 97 to 183 per year and was significantly higher than that of *E. coli* ESBL(+) (each year *p* < 0.001). Moreover, it has been steadily increasing. The highest number of infections caused by *E. coli* ESBL(+) occurred in 2018. The highest number of infections caused by *K. pneumoniae* ESBL(+) occurred in 2021. At 132, the number of ESBL(−) strains isolated from patients was the lowest in 2020. In other years, it remained at 169–223, much lower than for *E. coli* ESBL(−) (each year *p* < 0.001) (Figure 1 and Figure 11).

**Figure 12:**
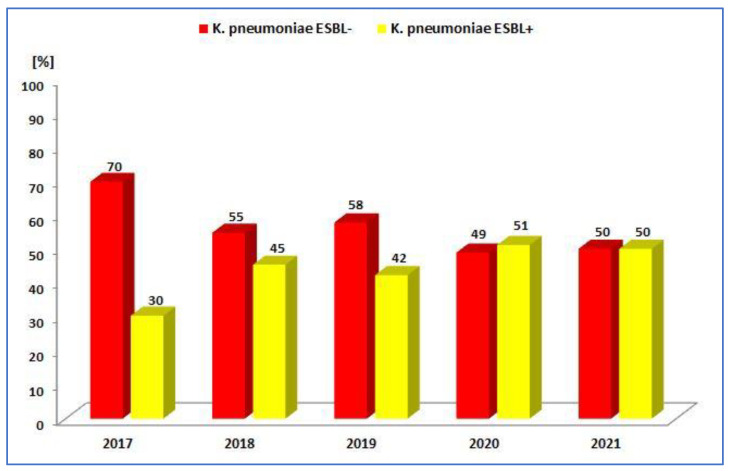
Comparison of the percentage of *K. pneumoniae* ESBL(+) and ESBL(−) strains from 2017 to 2021.

The percentage of *K. pneumoniae* ESBL(−) strains has been gradually decreasing and has ranged from 70% to 49% over the past five years, but there has been an increase in the percentage of ESBL(+) strains (from 30% to 51%) (Figure 12, Table 4). The year 2020 was the only year in which the percentage of β-lactamase-producing strains prevailed. It can be noted that each year, the percentage of *K. pneumoniae* ESBL(+) strains was much higher than that of *E. coli* ESBL(+); the difference is significant (2017 *p* < 0.001, 2018 *p* < 0.05, 2019 *p* < 0.001, 2020 *p* < 0.001, 2021 *p* < 0.001).

**Figure 13:**
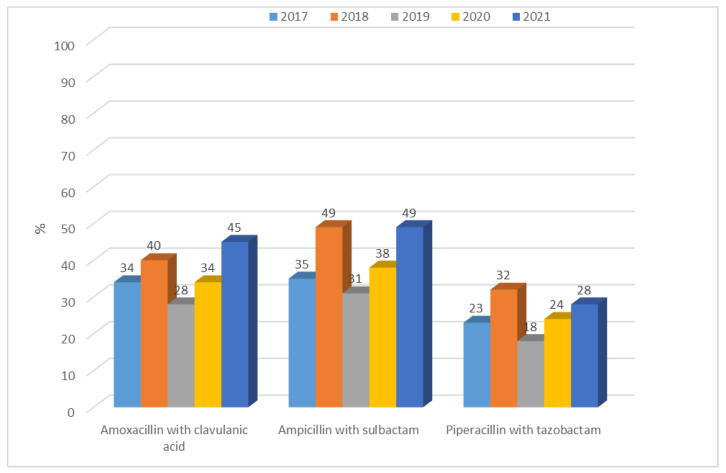
Percentage of *K. pneumoniae* ESBL(−) resistant strains with inhibitor penicillins.

**Figure 14:**
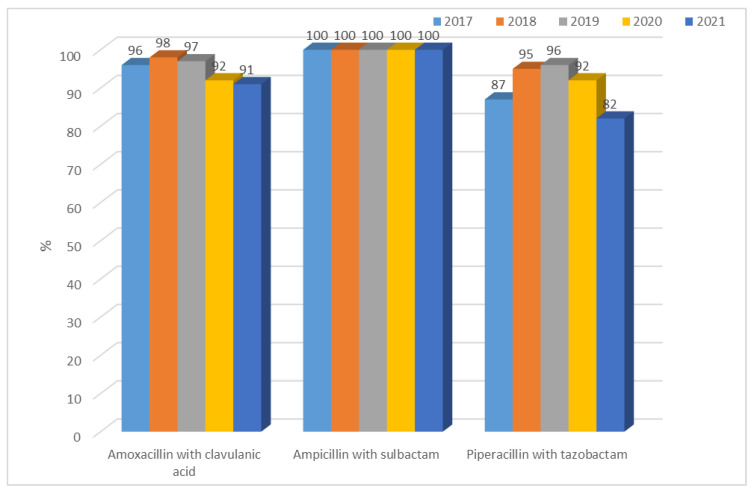
Percentage of *K. pneumoniae* ESBL(+) strains resistant to penicillins with inhibitors.

Resistance to penicillins with inhibitors for ESBL(−) strains was at a constant average level and ranged from 28% (n = 49; 2019) to 45% (n = 76; 2021) for amoxicillin with clavulanic acid and from 31% (n = 54; 2019) to 49% (n = 108; 2018 and 2021) for ampicillin with sulbactam. The differences between years were significant (amoxicillin with clavulanic acid *p* < 0.5, ampicillin with sulbactam *p* < 0.05, piperacillin with tazobactam *p* < 0.05). The highest susceptibility was observed for piperacillin with tazobactam, as in *E. coli* ESBL(+), and resistance has steadily increased over the past two years (Figure 13). There was a significant difference in 2021 compared with previous years: 2018, 2019, 2020 (*p* < 0.05). In the case of *K. pneumoniae*, however, susceptibility to ampicillin was not tested due to the natural resistance of these bacteria to this antibiotic. On the other hand, strains producing extended-spectrum β-lactamases showed greater resistance to penicillins with inhibitors than ESBL(−) strains, ranging from 82% to as much as 100% (each year significant *p* < 0.001, Figure 14). The differences between years were significant (amoxicillin with clavulanic acid *p* < 0.001, ampicillin with sulbactam *p* < 0.001, piperacillin with tazobactam *p* < 0.001).

**Figure 15:**
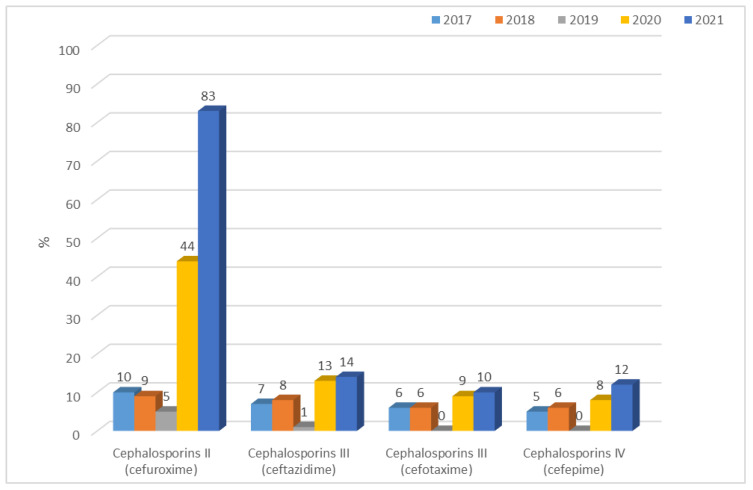
Percentage of cephalosporin-resistant *K. pneumoniae* ESBL(−) strains.

**Figure 16:**
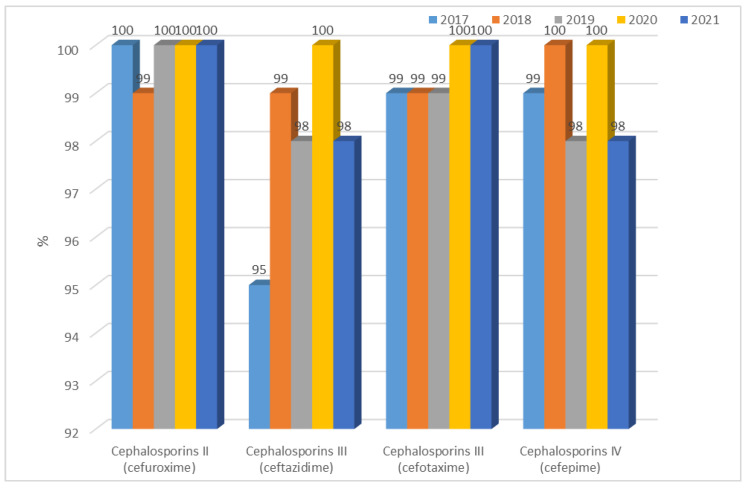
Percentage of *K. pneumoniae* ESBL(+) strains resistant to cephalosporins.

There are significant differences between the resistance of *K. pneumoniae* ESBL(−) strains in year-to-year analysis for each cephalosporin (*p* < 0.001). Until 2019, the percentage of *K. pneumoniae* ESBL(−) strains resistant to second-generation cephalosporins was at a consistently low level, ranging from 5% (n = 11) to 10% (n = 22). In the last two years, this resistance increased to 83% (n = 140, *p* < 0.001). A similar increase also occurred for *E. coli* ESBL(−). Among the third- and fourth-generation cephalosporins, the percentage of resistant strains that did not produce β-lactamases was relatively low, ranging from 1% to 14%, but this has gradually increased between 2020 and 2021 (Figure 15), comparing 2019 with 2020 and 2021 differences as significant for cefuroxime (2020 and 2021 *p* < 0.001), ceftazidime and cefotaxime (2020 *p* < 0.01, 2021 *p* < 0.001), and cefepime (2020 *p* < 0.05, 2021 *p* < 0.001). *K. pneumoniae* ESBL(+) strains consistently demonstrated very high resistance to second-, third-, and fourth-generation cephalosporins, ranging from 95% to 100% (Figure 16).

**Figure 17:**
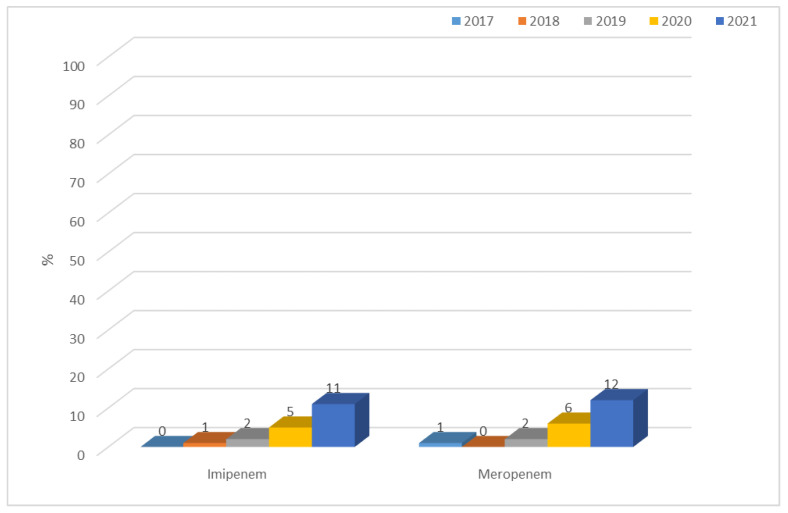
Percentage of *K. pneumoniae* strains resistant to carbapenems.

**Figure 18:**
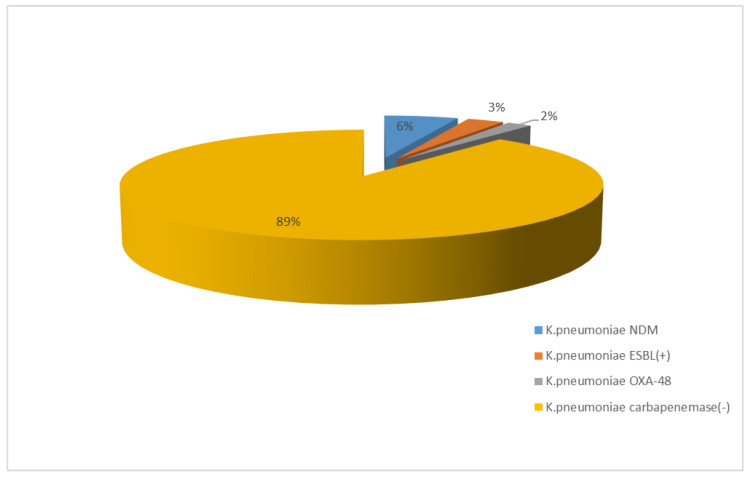
Comparison of the percentage of *K. pneumoniae* strains susceptible and resistant to carbapenems in 2021.

Over the past three years, an increase was observed in the resistance of β-lactamase-producing strains to carbapenems, which was up to 12% for meropenem. The highest percentage of resistant strains was recorded for 2021. During that year, NDM strains (6%) predominated among carbapenem-resistant bacilli. One hundred per cent of ESBL(−) strains at the selected facility showed susceptibility to imipenem and meropenem (Figure 17 and Figure 18).

**Figure 19:**
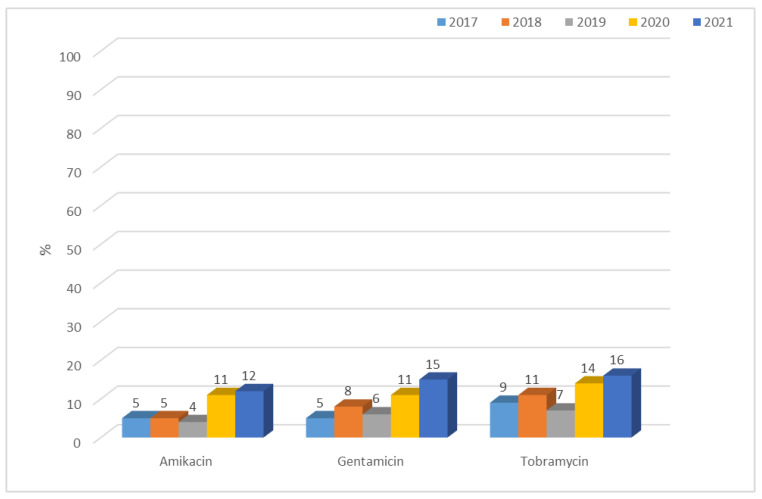
Percentage of *K. pneumoniae* ESBL(−) strains resistant to aminoglycosides.

**Figure 20:**
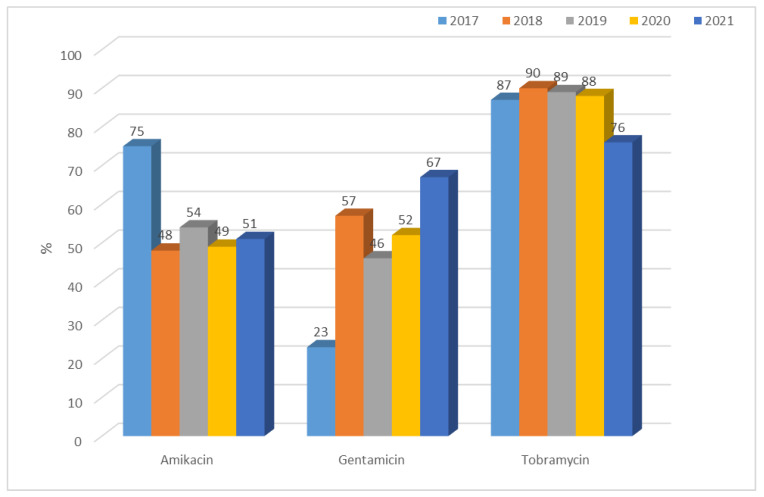
Percentage of *K. pneumoniae* ESBL(+) strains resistant to aminoglycosides.

Resistance of *Klebsiella* ESBL(−) strains to aminoglycosides was low, ranging from 4% to 12% for amikacin, 5% to 15% for gentamicin, and 7% to 16% for tobramycin. A gradual increase was observed over the past two years in the resistance of these bacilli to aminoglycosides (Figure 19). Differences in resistance for amikacin and for gentamicin between particular years were significant (for both *p* < 0.01). Among ESBL(+) strains, higher resistance was observed, ranging from 48% (n = 88; 2018) to 75% (n = 73; 2017) for amikacin, from 23% (n = 22; 2017) to 67% (n = 113; 2021) for gentamicin, and from 76% (n = 128; 2021) to 90% (n = 165; 2018) for tobramycin (Figure 20). Differences in resistance comparing year-to-year were significant for amikacin and gentamicin *p* < 0.001, and for tobramycin *p* < 0.01. Differences in resistance comparing year-to-year were significant: for amikacin and gentamicine, resistance noticed in 2017 differs from each other year (*p* < 0.01, *p* < 0.001-depends on year), for tobramycin, 2021 differs significantly from 2018 and 2019 (*p* < 0.01). The resistance to aminoglycosides among both ESBL(+) and ESBL(−) *Klebsiella* strains was higher than that of *E. coli* strains.

**Figure 21:**
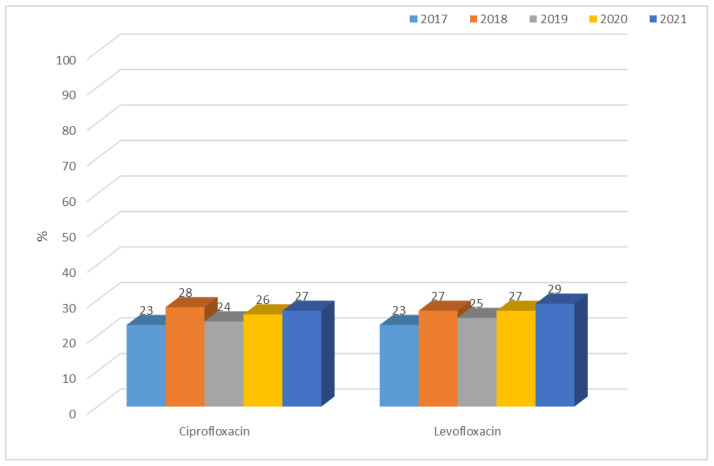
Percentage of *K. pneumoniae* ESBL(−) strains resistant to quinolones.

**Figure 22:**
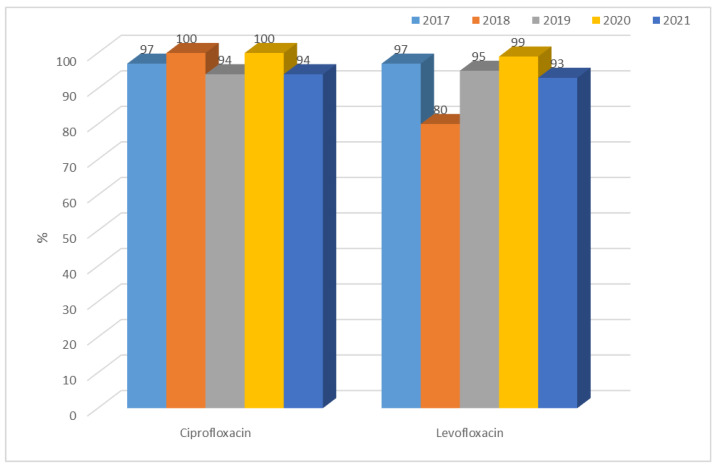
Percentage of *K. pneumoniae* ESBL(+) strains resistant to quinolones.

*K. pneumoniae* ESBL(−) strains showed consistent average resistance to quinolones of 23% to 28% for ciprofloxacin and 23% to 29% for levofloxacin, with lower values than in *E. coli* ESBL(−). In ESBL(+) bacilli, on the other hand, almost 100% resistance to quinolones was observed (Figure 21 and Figure 22).

### 3.2. Consumption of Antibiotics in the Hospital in the Studied Five-Year Period (in DDD/100 Patient Days)

The table above (Table 5) shows that the highest use of antibiotics across the hospital took place in 2021 (60.5 DDD/100 patient days) and the lowest in 2019 (34.7 DDD/100 patient days). In 2017 and 2018, second-generation cephalosporins and quinolones were the most frequently used group of antibiotics; in 2019 and 2021, it was penicillins with inhibitors, and; in 2020, it was third-generation cephalosporins and quinolones. In contrast, the use of tetracyclines, penicillins (although an increase in their use can be observed in the last year), fourth-generation cephalosporins, carbapenems, macrolides, lincosamides, aminoglycosides, glycopeptides, and polymyxins is at a relatively constant low level.

**Figure 23:**
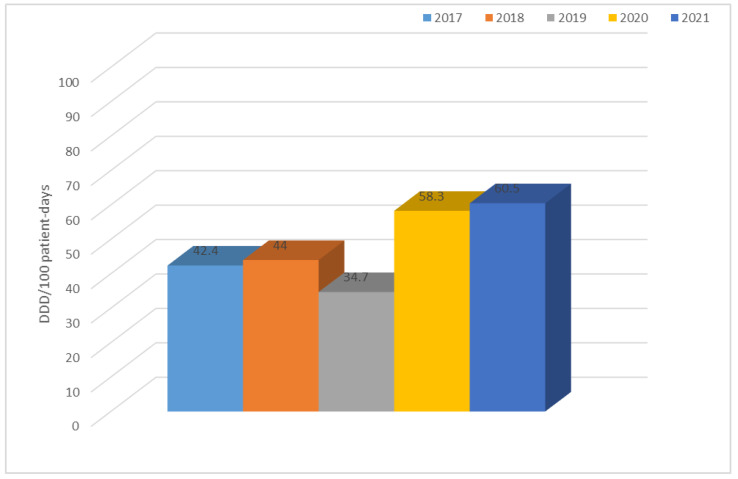
Consumption of antibiotics in all departments of the hospital over the past five years.

Figure 23 illustrates the use of all antibiotics across the hospital. The data shows that antibiotic use varied from 34.7 DDD/100 patient days in 2019 to 60.5 DDD/100 patient days in 2021. A significant increase was observed in the last two years.

**Figure 24:**
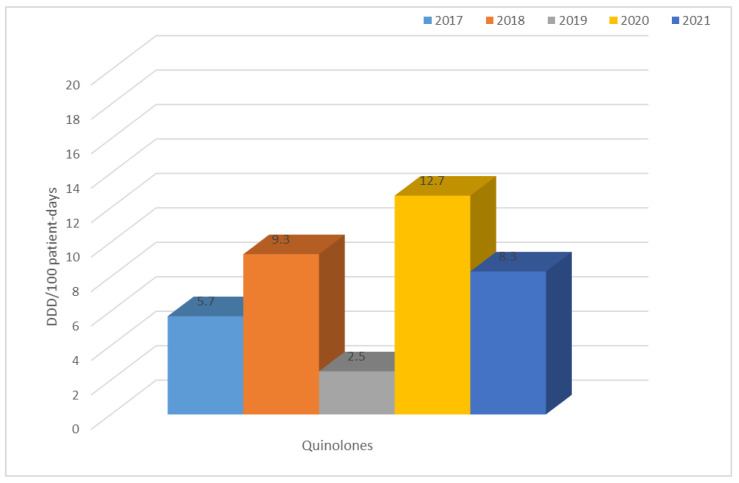
Consumption of quinolones (ciprofloxacin and levofloxacin) in 2017–2021.

Analysis of the use of antibiotics from different groups showed a clear increase in some of the groups. This is the case with quinolones, whose use is quite high in relation to other antibiotics, and has increased even more significantly in 2020 (Figure 24).

**Figure 25:**
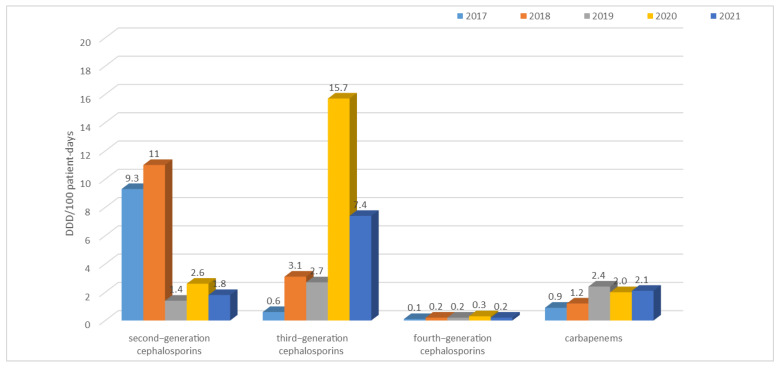
Consumption of second-generation cephalosporins (cefuroxime), third-generation cephalosporins (cefotaxime, ceftriaxone, ceftazidime), fourth-generation cephalosporins (cefepime), and carbapenems (imipenem, meropenem) in 2017–2021.

The situation is similar for third-generation cephalosporins (Figure 25), the use of which increased sharply in 2020 (reaching as much as 15.7/100 patient days), but in 2021, the level of their consumption was 7.4 DDD/100 patient days, which is still almost three times higher than in 2017–2019.

As the above analysis shows, the use of carbapenems also doubled between 2019 and 2021.

**Figure 26:**
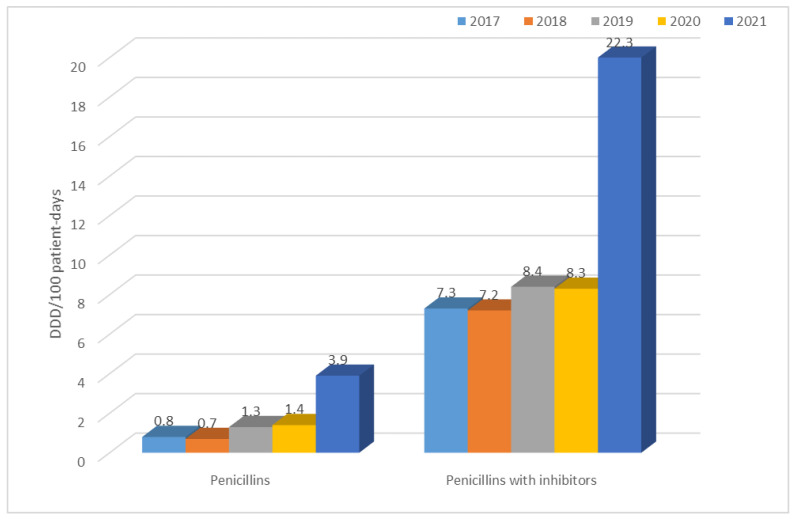
Consumption of penicillins (ampicillin) and penicillins with inhibitors (amoxicillin with clavulanic acid, ampicillin with sulbactam, and piperacillin with tazobactam) in 2017–2021.

A sharp, almost 3-fold increase in their use was also recorded in 2021 for penicillins with inhibitors (Figure 26).

The use of penicillins with inhibitors, on the other hand, was much higher than that of penicillins in each of the years analyzed, ranging from 7.2 DDD/100 patient days in 2018 to 22.3 DDD/100 patient days in 2021. In this case, there was also a sharp, almost 3-fold increase in their use in 2021.

For other antibiotics used in the treatment of *Enterobacteriaceae* infections, such as fourth-generation cephalosporins, aminoglycosides, tetracyclines, polymyxins, or cotrimoxazole, their use remained relatively low and no significant increase was found.

## 4. Discussion

The increasing antibiotic resistance of microorganisms observed in recent years is a serious problem for both humans and animals [28]. According to the WHO and the European Center for Disease Prevention and Control, it is one of the most serious medical challenges of the 21st century. It is believed that among the causes of the weakened bacterial response to antibiotics are improper use of antibiotics, administration of excessive amounts of antibiotics, discontinuation of therapy, or treatment with poorly selected groups of antibiotics, which accelerates the formation and spread of resistant clones [29]. At the same time, it is emphasized that although the process of acquiring resistance is a longstanding phenomenon in nature, it is much slower under natural conditions [30]. Bacterial resistance appears to be particularly dangerous for patients residing in healthcare facilities, which is why it is so important to control drug use and observe changes in the resistance of strains causing infections [31]. In Europe, an estimated 25,000 people died from bacterial sepsis caused by antibiotic-resistant bacteria in 2007, and by 2050, this number can reach as many as 10 million [32]. Nowadays, due to the lack of susceptibility to certain drugs, therapy is becoming much more complicated and expensive, and the results are not always effective. There are situations in which microbes are found to be insusceptible to antibiotics of the last resort [17]. There is also an increasing number of very serious infections whose etiologic agents are extramedullary pathogens, posing a challenge to modern medicine, both diagnostically and therapeutically [31,33]. It is worth noting that isolated bacteria often show acquired resistance to at least one antibiotic from a minimum of three different groups (MDR—Multidrug Resistant). There are also XDR (Extensively Drug Resistant) and PDR (Pandrug Resistant) strains, which means a resistance to at least one antibiotic of each group and insusceptibility to all available therapeutic substances, respectively [34]. Both *E. coli* and *K. pneumoniae* are among the most frequently detected bacterial carriers of clinically relevant drug resistance genes [9,35]. In 2017–2021 in Poland, the microorganism causing the highest number of nosocomial infections was *E. coli* [36,37,38,39,40]. In addition, in 2019, it was the most common etiologic factor in the development of sepsis in patients [38]. This situation is also reflected in the multi-profile hospital data analyzed in this paper, which confirm that between 2017 and 2021, this bacterium was the most common cause of infection. *E. coli* is the microorganism most frequently causing urinary tract infections and is one of the first multidrug-resistant bacteria to emerge in hospitals [41]. In recent years, in Poland, the resistance of this microorganism to aminopenicillins, fluoroquinolones, third-generation cephalosporins, and aminoglycosides has been at a higher level than the European average. For example, in 2017, the percentage of ampicillin-resistant strains was 69.4%, while in EU/EEA countries, it was 58.7% [36]. Based on our own results, the percentage of ampicillin-resistant strains was 59%, which was close to the European average and was significantly lower than the Polish average. However, the most important fact seems to be that over the past five years, susceptible strains (88–92%) that do not produce extended-spectrum enzymes were responsible for most infections occurring in the analyzed hospital. In contrast, the percentage of resistant strains ESBL(+) was at a constant low level (8–12%) and there was no upward trend in the share of these strains in infections.

The resistance of *E. coli* to fluoroquinolones, which in 2009–2012 in Poland was at the level of 23–29%, is also a problem. In the 2017–2019 period, the percentage was already between 33% and 35.9% (in EU/EEA countries, it was 25.7% on average), indicating its continuous increase and spread [36,37,38,39,40]. In China, on the other hand, more than 60% of strains showed resistance to antibiotics in this group, while in the United States, the percentage reached an average of 10% to 30% [42,43]. Based on our own data, we can conclude that the percentage of ciprofloxacin-resistant strains was 35% for ESBL(−) strains in 2019, which is in line with the Polish average [38]. It is also worth noting that in 2020–2021, the share of these strains in infections decreased to 26%, so it was close to the European average, which allowed us to conclude that, in the analyzed hospital, in the group of ESBL(−) strains, no build-up of antibiotic resistance was observed. These values were completely different in the case of *E. coli* ESBL(+) strains, where resistance to quinolones over the past five years reached almost 100% (81–91%). The analysis of nationwide data showed that in Poland, resistance to third-generation cephalosporins has increased in recent years, reaching 17.1% in 2019 and thus surpassing the European average (14.9%) [38]. In contrast, data from Asia indicates that the percentage was much higher, at around 60% for cefotaxime and 30% for ceftazidime [44]. The results obtained at the analyzed hospital indicate that among ESBL(−) strains, there was very low resistance to third-generation cephalosporins, amounting to a maximum of 3%. Noteworthy, however, is the observed dynamic increase in resistance to cefuroxime in 2020–2021, reaching 73%. ESBL(+) strains, on the other hand, showed almost 100% resistance to the second-, third-, and fourth-generation cephalosporins, which is obvious given the production of these enzymes.

After 2012, in Poland, *E. coli* strains also showed relatively constant resistance to aminoglycosides (at around 13%), while the European average ranged from 11% to 12% during that period [45]. In the case of the specialized hospital whose data were analyzed in this paper, the percentage of ESBL(−) aminoglycoside-resistant strains was lower than both the Polish and the European average, and reached a maximum of 8% for gentamicin in 2018. Among ESBL(+) strains, on the other hand, the percentage was unfortunately much higher, reaching up to 50% for tobramycin. In the case of *E. coli*, as well as in the cases of other *Enterobacteriaceae*, the most serious problem is the increasingly detected resistance to carbapenems [4,46]. Particularly dangerous are class A carbapenemases (KPC), class B (MBL), and class D (OXA-48), which determine resistance to all β-lactams used in medicine (MBLs in their substrate spectrum exclude only monobactams). Currently, the most commonly identified type of MBL in Poland is the NDM-1 enzyme, which was first isolated in 2011 [4,17]. According to EARS-Net data, in the case of *E. coli*, resistance to carbapenems was <1% in Europe and <0.1% in Poland, while in China, almost 2% of strains lack susceptibility to these antibiotics [37,44,47]. In the analyzed hospital, on the other hand, there were no *E. coli* bacteria that showed resistance to imipenem and meropenem in the last four years, while the situation was completely different for *K. pneumoniae* bacilli. In the case of carbapenem insusceptibility in these bacteria, the Polish average was 8% with a steady increase after 2016, likely due to the spread of clones producing carbapenem-hydrolyzing enzymes [46]. In Europe, the share of strains resistant to these antibiotics was estimated at 10% in 2020, while in Asian countries, the percentage was even higher, reaching up to 21% [48,49]. In the analyzed hospital, increasing resistance to this group of drugs was observed from 2019, amounting in 2021 to 12% for meropenem, which is above the national average. It was also found that the metallobetalactamases NDM and OXA-48 were mainly responsible for the lack of susceptibility to carbapenems in *K. pneumoniae* in 2021.

In Poland, while *E. coli* resistance to antibiotics seems to have been increasing in recent years, the analyses carried out in this paper show that in the hospital analyzed in this study, the data were different, as a lower proportion of ESBL(+) strains resistant to penicillins with inhibitors was observed, while their high susceptibility to piperacillin with tazobactam (70%-88% susceptibility) still persisted. After 2019, there was also a decline in the resistance of both ESBL(+) and ESBL(−) strains to aminoglycosides. In the case of *K. pneumoniae*, in contrast to *E. coli*, an increasing number of ESBL(+) strains was observed, which 2020 exceeded the number of strains not producing extended-spectrum β-lactamases in the analyzed hospital. This is confirmed by the high percentage of ESBL(+) strains resistant to penicillins with inhibitors and to cephalosporins, reaching 100%. Among ESBL(−) strains, resistance to ceftazidime and cefotaxime was showed by about 60% of them in Poland, and by about 50% in China, while in the analyzed hospital, resistance has been increasing over the past two years and has been at the level of 9–14% [36,37,44]. Resistance of *K. pneumoniae* ESBL(−) bacilli to second-generation cephalosporins also increased significantly between 2020 and 2021. Resistance of non-beta-lactamase-producing strains to penicillins with inhibitors was significantly lower compared with ESBL(+) isolates, but showed an increasing trend after 2019. The highest susceptibility of these bacteria was found for piperacillin with tazobactam (68–82%).

The percentage of *K. pneumoniae* strains resistant to aminoglycosides in the analyzed hospital was a maximum of 16% for ESBL(−) strains and 90% for ESBL(+) isolates, while the national average at the same time ranged from 47% to 55% [36,37,38,47]. It seems that the high resistance of strains producing extended-spectrum β-lactamases to this group of antibiotics (the highest to tobramycin) is due to the presence of the same plasmids of both genes encoding ESBL enzymes and aminoglycoside resistance genes [4].

On the other hand, in Poland, *Klebsiella* bacilli resistance to quinolones was quite high but relatively constant, ranging between 62% and 69% in 2017–2019 [36,37,38,39,40,44]. In the hospital referred to in this paper, the percentage was much higher, especially for ESBL(+) isolates. Lack of susceptibility to this group of antibiotics was shown by almost 100% of these strains. In the case of isolates that do not produce ESBL-type beta-lactamases, the percentage was almost twice lower than the national average. This may be related to the COVID-19 pandemic, during which selection of MDR clones occurred through overuse of multiple broad-spectrum antibiotics in patients infected with SARS-CoV-2, often without specific justification and without microbiological testing.

This was confirmed by an analysis of antibiotic use at the described hospital, which showed a large increase in the use of quinolones in 2020, especially in relation to the previous year (an increase from 2.5 to 12.7 DDD/100 patient days). The level of consumption of quinolones throughout the analyzed five-year period is also quite high in relation to other antibiotics. It can be concluded that, over the past five years, these antibiotics were among the most frequently used in the hospital in question. The situation is similar for third-generation cephalosporins. During the pandemic, most COVID-19 patients received ceftriaxone. The decline in susceptibility to these antibiotics can largely be attributed to their overuse. Additionally, the increase in carbapenem use in recent years is contributing to the selection of carbapenemase-producing strains, although this is undoubtedly a trend across Europe [48,49]. It is hoped that these analyses will increase the effectiveness of empirical therapy for suspected Gram-negative bacilli infections, based on the use of antibiotics for which the percentage of susceptible strains is the highest. They will certainly provide answers as to which antibiotics need to be avoided in hospital therapy in order to not further induce resistance. Undoubtedly, this research needs to be continued in order to track the development of resistance over the next few years, especially those following the COVID-19 pandemic.

## 5. Conclusions

1. *E. coli* was the microorganism responsible for the largest number of infections in the studied hospital, as in the country as a whole, but about 90% were strains susceptible to multiple antibiotics, and the percentage of resistant strains has not increased in the past five years;

2. The highest number of infections whose etiological agent was *K. pneumoniae* ESBL(+) and *E. coli* ESBL(+) was observed in 2018;

3. In the analyzed five-year period, the number of MDR *K. pneumoniae* strains increased successively, which seems to be related to increased use, especially in the pandemic period, of broad-spectrum antibiotics, mainly penicillins with inhibitors, third-generation cephalosporins, and carbapenems;

4. The analysis carried out will allow for an increase in the effectiveness of empirical therapy in the hospital and a more cautious use of antibiotics that leads to selection of MDR strains.

## Figures and Tables

**Table 1:** Number of tested strains of each microorganism isolated from infections in 2017–2021.

	2017	2018	2019	2020	2021
*Escherichia coli*	791 (71.2%)	917 (69.4%)	851 (73.7%)	742 (73.3%)	770 (69.5%)
*Klebsiella pneumoniae*	320 (28.8%)	404 (30.6%)	303 (26.3%)	270 (26.7%)	338 (30.5%)
Total	1111	1321	1154	1012	1108
*p*-Value	<0.001	<0.001	<0.001	<0.001	<0.001

**Table 2:** Antibiotics to which the susceptibility of the tested strains was determined.

Bacteria	Selected Antibiotics
*Escherichia coli*	Gentamicin, tobramycin, amikacin, levofloxacin, ciprofloxacin, ampicillin, amoxicillin/clavulanic acid, ampicillin/sulbactam, piperacillin/tazobactam, cefuroxime, ceftazidime, cefotaxime, cefepime, imipenem, meropenem
*Escherichia coli* ESBL(+)	Gentamicin, tobramycin, amikacin, levofloxacin, ciprofloxacin, amoxicillin/clavulanic acid, ampicillin/sulbactam, piperacillin/tazobactam, cefuroxime, ceftazidime, cefotaxime, cefepime, imipenem, meropenem
*Klebsiella pneumoniae*	Gentamicin, tobramycin, amikacin, ciprofloxacin, levofloxacin, amoxicillin/clavulanic acid, ampicillin/sulbactam, piperacillin/tazobactam, cefuroxime, ceftazidime, cefotaxime, cefepime, imipenem, meropenem
*Klebsiella pneumoniae* ESBL(+)	Gentamicin, tobramycin, amikacin, ciprofloxacin, levofloxacin, meropenem, imipenem, amoxicillin/clavulanic acid, ampicillin/sulbactam, piperacillin/tazobactam, cefuroxime, ceftazidime, cefotaxime, ceftriaxone, cefepime, imipenem, meropenem, colistin

**Table 3:** Number of *E. coli* ESBL(+) and ESBL(−) strains in 2017–2021.

	2017	2018	2019	2020	2021
ESBL(−)	729 (92.1%)	803 (87.6%)	768 (90.2%)	658 (88.7%)	698 (90.6%)
ESBL(+)	62 (7.9%)	114 (12.4%)	83 (9.8%)	84 (11.3%)	72 (9.4%)
Total	791	917	851	742	770
*p*-Value	<0.001	<0.001	<0.001	<0.001	<0.001

**Table 4:** Number of *K. pneumoniae* ESBL(+) and ESBL(−) strains in 2017–2021.

	2017	2018	2019	2020	2021
ESBL−	223 (69.7%)	221 (72.7%)	175 (57.8%)	132 (48.9%)	169 (50.0%)
ESBL+	97 (30.3%)	83 (23.3%)	128 (42.2%)	138 (51.1%)	169 (50.0%)
Total	320	304	303	270	338
*p*-Value	<0.001	<0.001	<0.05	>0.05	>0.05

**Table 5:** Consumption of antibiotics and chemotherapeutics for general use in 2017–2021 at the multi-profile hospital in Wroclaw.

	Antibiotic Consumption [DDD/100 Person-Days].													
Year	TET	PES	PES + in.	C II	C III	C IV	KARB	MAK	LINK	AM	CH	GP	POL	Total
2017	1.0	0.8	7.3	9.3	2.6	0.1	0.9	3.4	0.2	0.8	5.7	0.6	0.3	42.4
2018	0.6	0.7	7.2	11.0	3.1	0.2	1.2	0.6	0.6	0.6	9.3	0.9	0.4	44.0
2019	2.4	1.3	8.4	1.4	2.7	0.2	2.4	2.4	0.8	0.7	2.5	1.3	0.2	34.7
2020	0.8	1.4	8.3	2.6	15.7	0.3	2.0	0.6	1.1	1.0	12.7	1.1	0.4	58.3
2021	0.7	3.9	22.3	1.8	7.4	0.2	2.1	0.8	1.0	1.0	8.3	1.4	1.8	60.5

TET—tetracyclines, PES—penicillins, PES + in.—penicillins with inhibitors, C II—second-generation cephalosporins, C III—third-generation cephalosporins, C IV—fourth-generation cephalosporins, KARB—carbapenems, MAK—macrolides, AM—aminoglycosides, CH—quinolones, GP—glycopeptides, POL—polymyxins.

## Data Availability

Not applicable.
